# Improved Cerebral Time-of-Flight Magnetic Resonance Angiography at 7 Tesla – Feasibility Study and Preliminary Results Using Optimized Venous Saturation Pulses

**DOI:** 10.1371/journal.pone.0106697

**Published:** 2014-09-18

**Authors:** Karsten H. Wrede, Sören Johst, Philipp Dammann, Neriman Özkan, Christoph Mönninghoff, Markus Kraemer, Stefan Maderwald, Mark E. Ladd, Ulrich Sure, Lale Umutlu, Marc Schlamann

**Affiliations:** 1 Erwin L. Hahn Institute for Magnetic Resonance Imaging, University Duisburg-Essen, Essen, Germany; 2 Department of Neurosurgery, University Hospital Essen, Essen, Germany; 3 Department of Diagnostic and Interventional Radiology and Neuroradiology, University Hospital Essen, Essen, Germany; 4 Department of Neurology, Alfried Krupp von Bohlen und Halbach Hospital, Essen, Germany; 5 Division of Medical Physics in Radiology (E020), German Cancer Research Center (DKFZ), Heidelberg, Germany; University of Washington School of Medicine, United States of America

## Abstract

**Purpose:**

Conventional saturation pulses cannot be used for 7 Tesla ultra-high-resolution time-of-flight magnetic resonance angiography (TOF MRA) due to specific absorption rate (SAR) limitations. We overcome these limitations by utilizing low flip angle, variable rate selective excitation (VERSE) algorithm saturation pulses.

**Material and Methods:**

Twenty-five neurosurgical patients (male n = 8, female n = 17; average age 49.64 years; range 26–70 years) with different intracranial vascular pathologies were enrolled in this trial. All patients were examined with a 7 Tesla (Magnetom 7 T, Siemens) whole body scanner system utilizing a dedicated 32-channel head coil. For venous saturation pulses a 35° flip angle was applied. Two neuroradiologists evaluated the delineation of arterial vessels in the Circle of Willis, delineation of vascular pathologies, presence of artifacts, vessel-tissue contrast and overall image quality of TOF MRA scans in consensus on a five-point scale. Normalized signal intensities in the confluence of venous sinuses, M1 segment of left middle cerebral artery and adjacent gray matter were measured and vessel-tissue contrasts were calculated.

**Results:**

Ratings for the majority of patients ranged between good and excellent for most of the evaluated features. Venous saturation was sufficient for all cases with minor artifacts in arteriovenous malformations and arteriovenous fistulas. Quantitative signal intensity measurements showed high vessel-tissue contrast for confluence of venous sinuses, M1 segment of left middle cerebral artery and adjacent gray matter.

**Conclusion:**

The use of novel low flip angle VERSE algorithm pulses for saturation of venous vessels can overcome SAR limitations in 7 Tesla ultra-high-resolution TOF MRA. Our protocol is suitable for clinical application with excellent image quality for delineation of various intracranial vascular pathologies.

## Introduction

In recent years, time-of-flight magnetic resonance angiography (TOF MRA) [1] has become an important diagnostic tool to depict cerebral vasculature [2]–[4]. This non-invasive technique is now widely considered equivalent to the gold standard, digital subtraction angiography for some applications such as follow-up of coiled cerebral aneurysms [3]–[7]. One important factor for the increasing relevance of TOF MRA has been the availability of higher magnetic field strengths. Better signal-to-noise ratio at 3 Tesla has already significantly improved the spatial resolution compared to 1.5 Tesla [8]–[13], and 7 Tesla has pushed spatial resolution to the sub-millimeter scale [8], [9],[14]–[18] while maintaining reasonable acquisition times.

Ultra-high-field magnetic resonance imaging (MRI) at 7 Tesla is currently being introduced in clinical application [19]–[22]. Many technical difficulties have been overcome already, but several restricting issues have still to be resolved. One major limitation is imposed by specific absorption rate (SAR) restriction. To unleash the full potential of 7 Tesla MRI, modified pulse sequences have to be employed. Such sequences with special spatial presaturation pulses were first described by Felmlee [23], and their implementation, application, and clinical relevance have been demonstrated in several subsequent publications [1]–[3], [24], [25]. SAR limitations restrict conventional saturation pulses at 7 Tesla, due to the fact that these limits lead to unreasonable extension of acquisition time or suboptimal image contrast based on lowered flip angles of imaging excitation pulses. This study presents a protocol for ultra-high-resolution TOF MRA suitable for clinical application.

## Materials and Methods

### Ethics Statement

The study was conducted according to the principles expressed in the Declaration of Helsinki and was approved by the local university institutional review board (Ethik-Kommission der medizinischen Fakultät der Universität Duisburg-Essen). Written informed consent was obtained before each examination.

### Study Design and Population

This prospective study evaluated the diagnostic ability of high-resolution 7 Tesla TOF MRA for various intracranial vascular pathologies (aneurysms, AVM, AVF, Moya-Moya). The study group comprised of 25 neurosurgical patients (male n = 8, female n = 17; average age 49.64 years; range 26–70 years). Inclusion criteria were: 1) single or multiple intracranial vascular pathologies, 2) age 18–80 years, 3) ability to give informed consent and 4) legal competence. Exclusion criteria were: 1) monitoring on intensive care or intermediate care unit 2) cardiac pacemakers or any other electronic implants, 3) metallic implants, 4) pregnancy or breast feeding period, 5) claustrophobia and 6) chronic or episodic vertigo.

### Scanner and Coil System

All images were acquired on a 7 Tesla whole-body MRI system (Magnetom 7 T, Siemens Healthcare, Erlangen, Germany) equipped with a 32-channel Rx/Tx head coil (Nova Medical, Wilmington, MA, USA). The scanner has a gradient system of 45 mT/m maximum amplitude and a slew rate of 200 mT/m/ms.

### Examination at 7 Tesla

**Figure 1:**
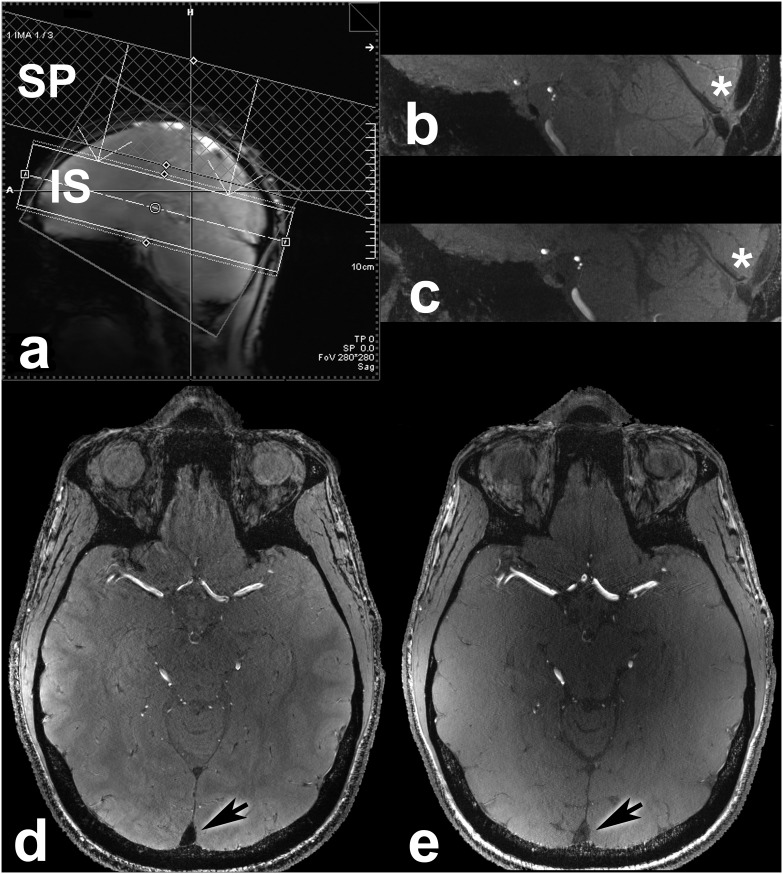
Shown is the localizer scan (a) of a 51-year-old male patient (subject 24) with a large arteriovenous malformation in the left hemisphere with thickness of TOF MRA imaging slab (IS) and saturation pulse (SP) illustrated. In a 29-year-old healthy subject a TOF MRA slab with identical coverage and spatial resolution was acquired with the non-optimized sequence in 17∶48 min (**b**: sagittal view, **d**: transverse view). With the optimized sequence the identical slab was acquired in 6∶22 min (**c**: sagittal view, **e**: transverse view). The same representative slice is shown for both sequences for direct comparison. In the optimized sequence TE could be reduced from 6.62 ms to 4.34 ms (due to a different asymmetric echo factor) and TR could be reduced from 56 ms to 20 ms (mainly due to reduced SAR) leading to better image contrast.

Prior to sequence acquisition, B_0_ shimming was performed using a vendor-provided gradient echo sequence and an algorithm based on the work of Schar [26]. A vendor-provided spin-echo sequence was used for B_1_ field mapping and local flip angle optimization. Following slice-selective excitation, two refocusing pulses sequentially generate a spin-echo and a stimulated echo. Transmitter adjustment volume was placed in the center of the brain leading to an average flip angle variation of less than 20% in the superior sagittal sinus. Spatial coverage of the TOF MRA slab and the saturation pulse is demonstrated on a sagittal localizer scan of a 40-year-old male patient suffering from a large right parietal AVM (Figure 1). Time-averaged local SAR in Watts/kg was assessed for every scan. The values were measured by the SAR supervision system of the MR scanner and read out from the DICOM header of the acquired imaging data.

### TOF MRA Sequence

The TOF MRA sequence is based on a 3D FLASH sequence with flow compensation and tilt-optimized non-saturated excitation (TONE) across the slab [27]. Datasets were acquired with an excitation flip angle of α = 18°, TE = 4.34 ms, TR = 20 ms, FOV 200 mm×169 mm×46 mm, 112 slices per slab (oversampling 14%), GRAPPA acceleration factor R = 4 (phase direction), partial Fourier 6/8 in both slice and phase directions, matrix of 896×756 (non-interpolated), and a voxel size of 0.22×0.22×0.41 mm^3^ in a total acquisition time of 6 min 22 s. For a shorter echo time, compared to the standard protocol, a different factor for the asymmetric echo was implemented in the modified protocol: 0.2 instead of 0.3 (ratio of columns acquired before and after echo). The variable-rate selective excitation (VERSE) algorithm [28] was used to reduce SAR contribution of excitation and venous saturation RF pulses [29]. Cutoff thresholds (percentage of maximum amplitude of the original pulse at which the pulse is cropped by the VERSE algorithm) of 50%/30% were chosen for excitation/saturation. The resulting duration was 0.6 ms/2 ms for excitation/saturation RF pulse (compared to 1 ms/3.8 ms in the unmodified standard protocol). A lower VERSE cutoff threshold for the saturation pulses than for the excitation pulses can be chosen because an exact saturation slice profile is not important as long as the saturation area is not overlapping with the imaging slice. The flip angle of the saturation RF pulses was additionally reduced (α_SAT_ = 35° instead of 90° which is normally used) to face SAR constraints [27].

### Venous Saturation Pulse

Modified saturation pulses were used to suppress the venous system within the slab and above including the superior sagittal sinus. To reduce total measurement time, the RF pulse and the following spoiler gradient were made shorter (2.3 ms [chosen duration before VERSE was applied]/4 ms instead of 4 ms/11 ms). To reduce SAR of the RF pulses, the VERSE algorithm was used on the saturation pulses with a 30% cut-off threshold. The flip angle, α_SAT_, of the saturation pulses was set to nominally 35° (variation of less than 20% in the superior sagittal sinus due to B_1_ inhomogeneity) as suggested in our previously published paper [27] to allow high saturation without exceeding the SAR limitations.

### Image Evaluation

Image evaluation was performed in consensus by two experienced neuroradiologists on standard post-processing Picture Archiving and Communcation system workstations (Centricity RIS 4.0i, GE Healthcare, USA). Visual evaluation was performed on 3D image reconstructions and on the source images in axial orientation.

For qualitative analysis the following features were evaluated, utilizing a five-point scale (5 = excellent, 4 = good, 3 = moderate, 2 = poor, and 1 = non-diagnostic):

1. Delineation arterial vessels in Circle of Willis
2. Delineation of vascular pathologies
3. Presence of artifacts
4. Vessel tissue contrast
5. Overall image quality

Prior to quantitative measurements, signal intensities of all scans were normalized. The image histogram of each subject was stretched, and shifted to match the baseline histogram of subject 1. Although this method does not correct for intra-subject B_1_ inhomogeneities, inter-scan variability of signal intensities caused by e.g. different transmitter voltage, head size and other factors is successfully minimized [30]. The histogram normalization was performed using the inormalize tool by Zijdenbos (version 1.5.1 for OS X as part of Medical Imaging NetCDF toolbox - MINC). MINC is a medical imaging data format and associated set of tools and libraries developed at the Montreal Neurological Institute and freely available online (http://www.bic.mni.mcgill.ca/ServicesSoftware). After preprocessing quantitative signal intensity measurements were then performed by region-of-interest (ROI) analysis using the interactive display tool “fslview” (version 3.1.8 for OS X, http://www.fmrib.ox.ac.uk/fsl/fslview). Vessel-tissue contrast ratio 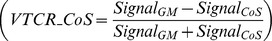 of the confluence of sinuses (CoS) and 
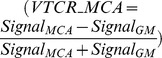
 middle cerebral artery (MCA) were assessed in correlation to surrounding gray matter (GM). Therefore, ROIs were placed in the largest diameter of the CoS 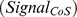 and proximal left M1 segments 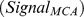 as well as adjacent gray matter 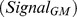. Diameters for ROIs slightly varied between subjects to account for anatomical variations and to maximize ROI size without including voxels from other tissue classes. The average diameter for the ROI of the CoS was 8–12 mm; the ROI for MCA was 3–5 mm in diameter; the ROI for brain parenchyma amounted to approximately 10 mm. In surgically treated Moyamoya patients ROIs were placed in the superficial temporal artery to middle cerebral artery bypass instead of the proximal M1 segment. Sufficient venous saturation in the confluence of sinuses was achieved by definition, if signal intensities in CoS were lower than in GM (VTCR_CoS>0). Arterial vessel tissue contrast was rated subjectively by both raters with focus on local angioarchitecture (VTCR_MCA>0.2 was defined as sufficient).

Differences of continuous scaled variables were tested by Student's t-test. For every mean value the standard error of mean (SE, 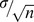) was calculated as an estimate of the population mean. Statistical analysis was carried out with the STATA software package (Stata/SE 12.1 for Mac (64-bit Intel), StataCorp, 4905 Lakeway Drive, College Station, Texas 77845 USA).

## Results

**Figure 2:**
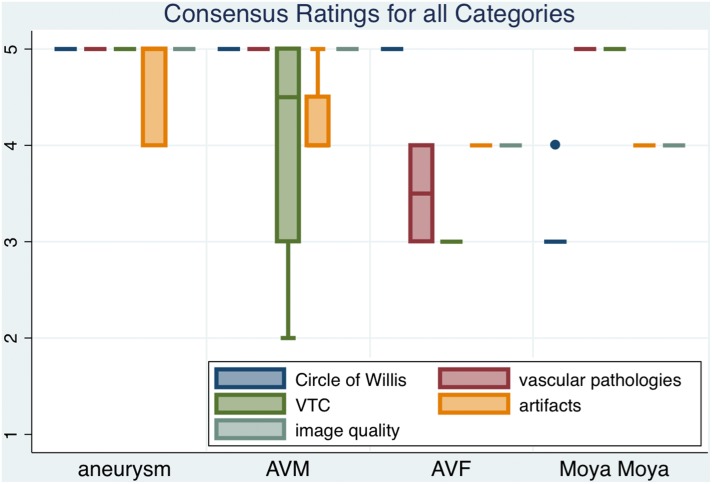
This figure shows the boxplots for all pathologies (aneurysms, AVM, AVF and Moyamoya angiopathy) and all rated categories. Delineation of Circle of Willis was rated excellent except for Moyamoya angiopathy, mainly due to the disease specific early ramifications of the middle cerebral artery branches. Delineation of vascular pathologies was rated excellent except for AVF. The complex angioarchitecture and close relationship to the skull bone complicate depiction in this pathology. Vessel tissue contrast was excellent in aneurysms and Moyamoya angiopathy, but poor to excellent in AVM and AVF. Arterialized hyperintense venous blood alters vessel tissue contrast in these patients. Presence of artifacts was rated mainly good to excellent for all pathologies and only pulsation artifacts led to lower ratings. VERSE specific artifacts did not significantly alter the images. Overall image quality was good to excellent for all pathologies.

All 25 neurosurgical patients tolerated the examination well and were successfully examined without exceeding SAR limitations (time averaged applied RF power in Watts/kg 8.493, range 6.077–9.988, SE 0.241) using 35° flip angles for saturation pulses. Table 1 gives an overview of basic demographic data and time-averaged local SAR in Watts/kg for every scan. Table 2 lists detailed information about consensus ratings for vessel and pathology delineation, vessel-tissue contrast, artifacts and overall image quality. A lower rating for presence of artifacts in some of the patients was solely due to pulsation artifacts. Slight aliasing artifacts were observable only in the uppermost/lowermost 2–3 slices in the axial images, which did not impair evaluation by the raters. Further VERSE specific artifacts (e.g. slice profile distortions or slice shifts especially for off-resonant spins) were not observed. Ratings were good to excellent for the majority of patients and pathologies. Figure 2 presents the ratings in boxplots for all pathologies and all rated categories. Table 3 shows detailed information on ROI mean signal intensity in confluence of sinuses, middle cerebral artery M1 segment (superficial temporal artery to middle cerebral artery bypass in surgically treated patients) and adjacent gray matter. Calculations of vessel-tissue contrast ratios show sufficient vessel-tissue contrast for venous vessels, arterial vessels and adjacent gray matter for the majority of patients and pathologies. The applied venous saturation pulses resulted in vessel-tissue contrast ratios for all patients as follows: mean VTCR_CoS = 0.401 (range 0.108–0.591, SE 0.024); VTCR_MCA = 0.68 (range 0.598–0.802, SE 0.011). The applied venous saturation pulses resulted in vessel-tissue contrast ratios for the patient subgroup with aneurysms and Moyamoya angiopathy as follows: mean VTCR_CoS = 0.44 (range 0.333–0.591, SE 0.015); VTCR_MCA = 0.674 (range 0.602–0.792, SE 0.012). The applied venous saturation pulses resulted in vessel-tissue contrast ratios for the patient subgroup with AVM and AVF as follows: mean VTCR_CoS = 0.279 (range 0.108–0.488, SE 0.073); VTCR_MCA = 0.697 (range 0.598–0.802, SE 0.032). Student’s t-test showed significantly higher VTCR_CoS (p = 0.003) for the patient subgroup with aneurysms and Moyamoya angiopathy compared to the subgroup of patients suffering of AVM or AVF.

**Table 1:** Basic demographic data for all patients and average SAR for TOF MRA sequence.

Subject	Sex	Age in Years	SAR	Pathology
1	female	52	9.988	aneurysm
2	female	26	9.116	aneurysm
3	female	56	9.088	aneurysm
5	female	56	8.166	aneurysm
7	male	70	9.47	aneurysm
9	male	45	9.035	aneurysm
12	female	66	9.351	aneurysm
13	female	52	8.666	aneurysm
14	female	54	6.657	aneurysm
15	female	69	6.264	aneurysm
16	female	51	6.077	aneurysm
17	female	60	6.684	aneurysm
21	female	53	9.7	aneurysm
4	male	40	9.955	AVM
22	male	49	9.363	AVM
23	male	40	7.701	AVM
24	male	51	8.031	AVM
11	male	58	7.306	AVF
25	male	61	7.228	AVF
6	female	35	9.658	Moyamoya
8	female	34	9.13	Moyamoya
10	female	29	7.911	Moyamoya
18	female	58	9.004	Moyamoya
19	female	34	9.088	Moyamoya
20	female	42	9.677	Moyamoya

TOF MRA: time-of-flight magnetic resonance angiography.

SAR: time-averaged local specific absorption rate; 10 Watts/kg allowed maximum.

**Table 2:** Consensus ratings for arterial vessel and pathology delineation, vessel-tissue contrast, artifacts and overall image quality.

Subject	Delineationof Circleof Willis	Delineationofvascularpathologies	Vessel-tissuecontrast	artifacts	imagequality	Pathology
1	5	5	5	5	5	aneurysm
2	5	5	5	4	4	aneurysm
3	5	5	5	4	5	aneurysm
5	5	5	5	4	5	aneurysm
7	5	5	5	5	5	aneurysm
9	5	5	5	5	5	aneurysm
12	5	5	5	5	5	aneurysm
13	5	5	5	4	5	aneurysm
14	5	5	5	5	5	aneurysm
15	5	5	5	4	5	aneurysm
16	5	5	5	5	5	aneurysm
17	5	5	5	5	5	aneurysm
21	5	5	5	5	5	aneurysm
4	5	5	2	4	5	AVM
22	5	5	4	4	5	AVM
23	5	5	5	5	5	AVM
24	5	5	5	4	5	AVM
11	5	3	3	4	4	AVF
25	5	4	3	4	4	AVF
6	3	5	5	4	4	Moyamoya
8	3	5	5	4	4	Moyamoya
10	3	5	5	4	4	Moyamoya
18	4	5	5	4	4	Moyamoya
19	3	5	5	4	4	Moyamoya
20	3	5	5	4	4	Moyamoya

For qualitative analysis images were evaluated, utilizing a five-point scale:

5 = excellent, 4 = good, 3 = moderate, 2 = poor, and 1 = non-diagnostic.

AVM: arteriovenous malformation AVF: dural arteriovenous fistula.

**Table 3:** Quantitative normalized signal intensity measurements.

Subject	CoS	GM	VTC_CoS	MCA	GM	VTC_MCA	Pathology
1	203	482	0.407	1270	244	0.678	aneurysm
2	217	470	0.368	1130	237	0.653	aneurysm
3	179	440	0.422	1082	228	0.652	aneurysm
5	150	479	0.523	1105	266	0.612	aneurysm
7	137	415	0.504	968	225	0.623	aneurysm
9	204	443	0.369	1195	225	0.683	aneurysm
12	168	430	0.438	1072	227	0.651	aneurysm
13	160	373	0.400	1221	212	0.704	aneurysm
14	107	416	0.591	1157	202	0.703	aneurysm
15	113	348	0.510	1117	196	0.701	aneurysm
16	158	479	0.504	1147	257	0.634	aneurysm
17	159	399	0.430	1084	221	0.661	aneurysm
21	110	274	0.427	862	154	0.697	aneurysm
4	429	533	0.108	1112	280	0.598	AVM
22	147	301	0.344	1080	152	0.753	AVM
23	90	251	0.472	1108	122	0.802	AVM
24	126	366	0.488	1074	211	0.672	AVM
11	347	461	0.141	1032	236	0.628	AVF
25	259	330	0.121	1168	184	0.728	AVF
6	162	336	0.349	1027	174	0.710	Moyamoya
8	108	261	0.415	1053	133	0.776	Moyamoya
10	147	449	0.507	986	245	0.602	Moyamoya
18	187	460	0.422	1004	220	0.641	Moyamoya
19	117	234	0.333	1007	117	0.792	Moyamoya
20	161	407	0.433	973	215	0.638	Moyamoya

Quantitative normalized signal intensity measurements were performed by region-of-interest (ROI) analysis.

Vessel-tissue contrast ratio 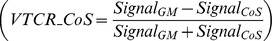 of the confluence of sinuses (CoS) and 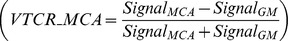
_._

middle cerebral artery (MCA) were assessed in correlation to surrounding gray matter (GM). Therefore, ROI were placed in the largest diameter of the CoS 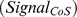 and proximal left M1 segments 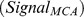 as well as adjacent gray matter 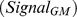.

The average diameter for the ROI of the CoS was 8–12****mm; the ROI for MCA was 3–5****mm in diameter; the ROI for brain parenchyma amounted to approximately 10****mm. In surgically treated Moyamoya patients ROI were placed in the superficial temporal artery to middle cerebral artery bypass instead of the proximal M1 segment.

AVM: arteriovenous malformation AVF: dural arteriovenous fistula.

CoS: confluence of sinuses SSS: superior sagittal sinus.

GM: gray matter MCA: middle cerebral artery M1 segment**.**

### Aneurysms

Thirteen patients with a total of 19 intra-cranial unruptured aneurysms were enrolled in the study (male n = 2, female n = 11; average age 54.61 years; range 26–70 years). Consensus ratings were excellent for delineation of Circle of Willis (mean 5.0, range 5–5, SE 0.0), excellent for vascular pathology delineation (mean 5.0, range 5–5, SE 0.0), excellent for vessel-tissue contrast (mean 5.0, range 5–5, SE 0.0), good for presence of artifacts (mean 4.69, range 4–5, SE 0.133) and excellent for overall image quality (mean 5.0, range 5–5, SE 0.0). The applied venous saturation pulses resulted in vessel-tissue contrast ratios as follows: mean VTCR_CoS = 0.45 (range 0.368–0.591, SE 0.019); VTCR_MCA = 0.667 (range 0.612–0.704, SE 0.009).

### Arteriovenous malformations

**Figure 3:**
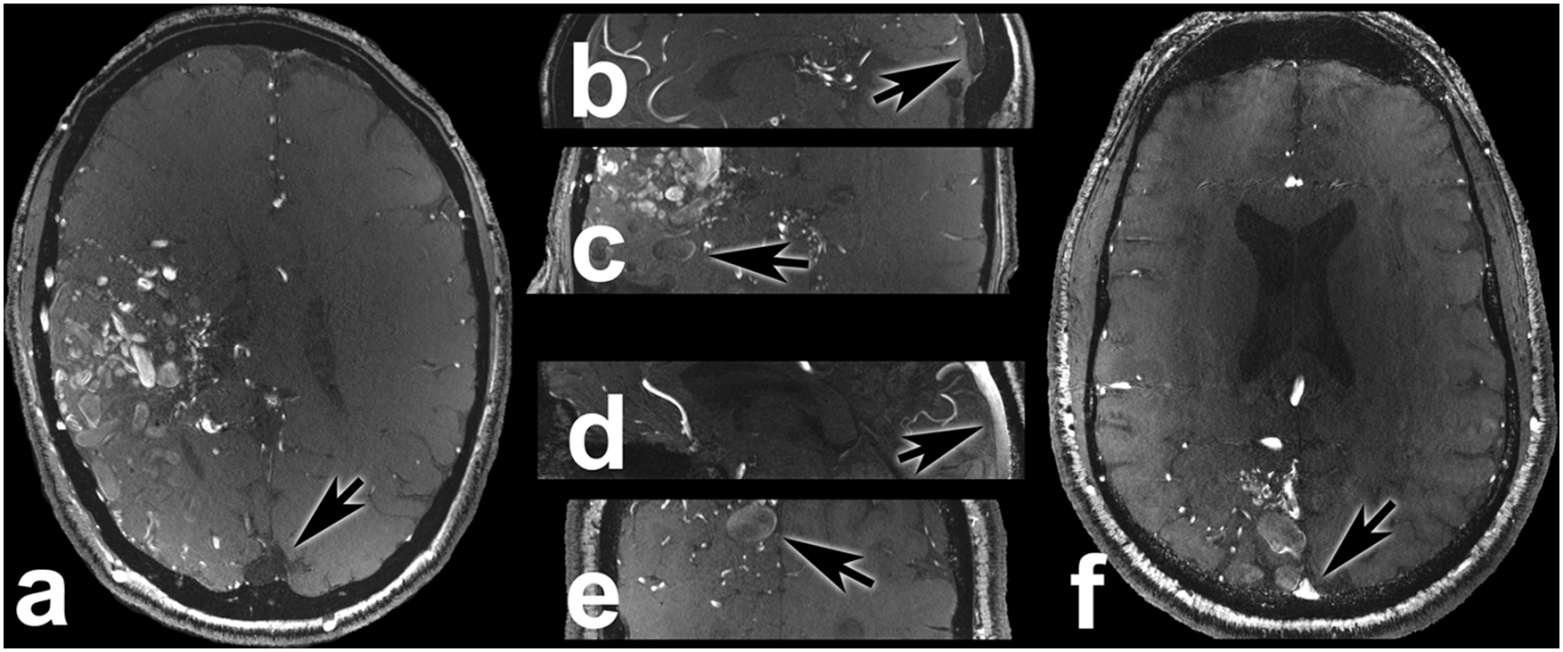
This 40-year-old male patient (a–c) (subject 23) suffered from a right hemispherical AVM (Spetzler-Martin [31] grade IV). Arrows are in transversal (**a**) and sagittal (**b**) views are indicating the saturated superior sagittal sinus. Arrow in coronal view (**c**) is marking a partially saturated draining vein. This 40-year-old male patient (**d**–**f**) (subject 4) suffered of a right parietal parasagittal fistulous AVM. Signal intensity in the upper part of the sagittal sinus next to the outlet of the draining vein was equal to signal intensity of left middle cerebral artery M1 segment (**f**): arrow in this transverse view is marking unsaturated superior sagittal sinus). In the further distal part of the superior sagittal sinus close to the confluence of sinuses, signal intensity is gradually decreasing until it is lower than in the neighboring occipital gray matter (**d**: arrow in this sagittal view is marking gradually saturated superior sagittal sinus). Arrow marks a partially saturated draining vein in coronal view (**e**). Depending on the individual cerebrovascular anatomy and venous drainage of the AVM, venous saturation can be altered significantly in parts of the TOF MRA imaging slab. Regardless of lower venous saturation in some cases overall image quality was nevertheless rated good to excellent.

Four patients with intracranial AVM were enrolled in the study (male n = 4, female n = 0; average age 45 years; range 40–51 years). Consensus ratings were excellent for delineation of Circle of Willis (mean 5.0, range 5–5, SE 0.0), excellent for vascular pathology delineation (mean 5.0, range 5–5, SE 0.0), good for vessel-tissue contrast (mean 4.0, range 2–5, SE 0.707), good for presence of artifacts (mean 4.25, range 4–5, SE 0.25) and excellent for overall image quality (mean 5.0, range 5–5, SE 0.0). The applied venous saturation pulses resulted in vessel-tissue contrast ratios as follows: mean VTCR_CoS = 0.353 (range 0.108–0.488, SE 0.088); VTCR_MCA = 0.706 (range 0.578–0.802, SE 0.045). Venous saturation was lower compared to other pathologies due to inherent features of AVMs, in particular the arterialized venous drainage. Decreasing volume of arterialized draining blood flowing through the saturation slab increases signal intensity. Patient 4 suffered of a right parietal parasagittal fistulous AVM. Signal intensity in the upper part of the sagittal sinus next to the outlet of the draining vein was equal to signal intensity of left middle cerebral artery M1 segment. In the confluence of venous sinuses signal intensity was lower than in the neighboring occipital gray matter (Figure 3 d, gradually decreasing signal intensity in sagittal view). Figure 3 b depicts a partially saturated draining vein in a 40-year-old male patient (subject 23) who suffered from a right hemispherical AVM (Spetzler-Martin [31] grade IV). Depending on the individual cerebrovascular anatomy and venous drainage of the AVM, venous saturation can be altered significantly in parts of the TOF MRA imaging slab. The specific features of AVM delineation in 7 Tesla TOF MRA scans are presented in transversal, sagittal and coronal views for subject 4 and 23.

### Moyamoya angiopathy

**Figure 4:**
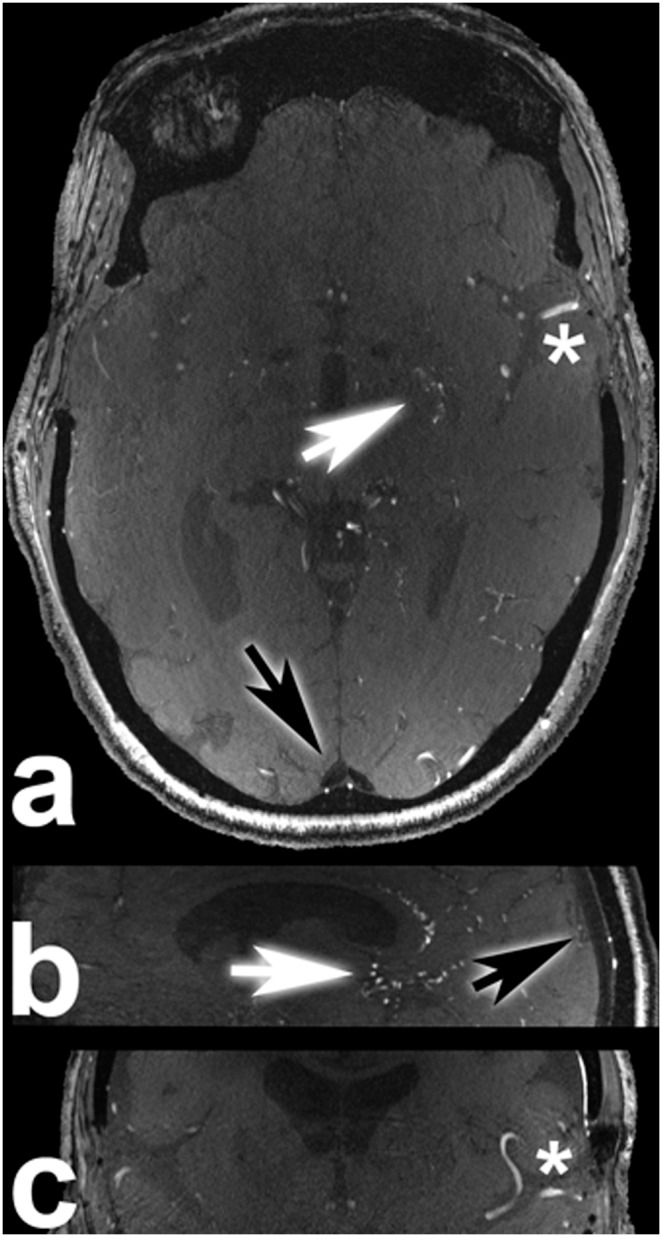
These are the transversal (a), sagittal (b) and coronal (c) views of a 42-year-old female Moyamoya patient (subject 20) after bilateral superficial temporal artery to middle cerebral artery bypass surgery (asterisks next to bypass, black arrows pointing on saturated superior sagittal sinus). The Moyamoya typical anomalously early ramification of middle cerebral artery branches are best depicted in axial and coronal views (white arrows in **a** and **b**).

Six patients suffering from Moyamoya angiopathy were enrolled in the study (male n = 0, female n = 6; average age 38.67 years; range 29–58 years). Consensus ratings were moderate for delineation of Circle of Willis (mean 3.167, range 3–4, SE 0.167), excellent for vascular pathology delineation (mean 5.0, range 5–5, SE 0.0), excellent for vessel-tissue contrast (mean 5.0, range 5–5, SE 0.0), good for presence of artifacts (mean 4.0, range 4–4, SE 0.0) and good for overall image quality (mean 4.0, range 4–4, SE 0.0). The inherent features of Moyamoya angiopathy with slim and sometimes completely blocked arteries in the Circle of Willis and complex collateral circulation especially in surgically treated patients resulted in only moderate ratings for delineation of the Circle of Willis. The applied venous saturation pulses resulted in vessel-tissue contrast ratios as follows: mean VTCR_CoS = 0.41 (range 0.333–0.507, SE 0.026); VTCR_MCA = 0.693 (range 0.602–0.792, SE 0.032). Figure 4 shows a scan of a 42 years-old female patient (subject 20) after bilateral superficial temporal artery to middle cerebral artery bypass surgery.

### Dural arteriovenous fistula (AVF)

**Figure 5:**
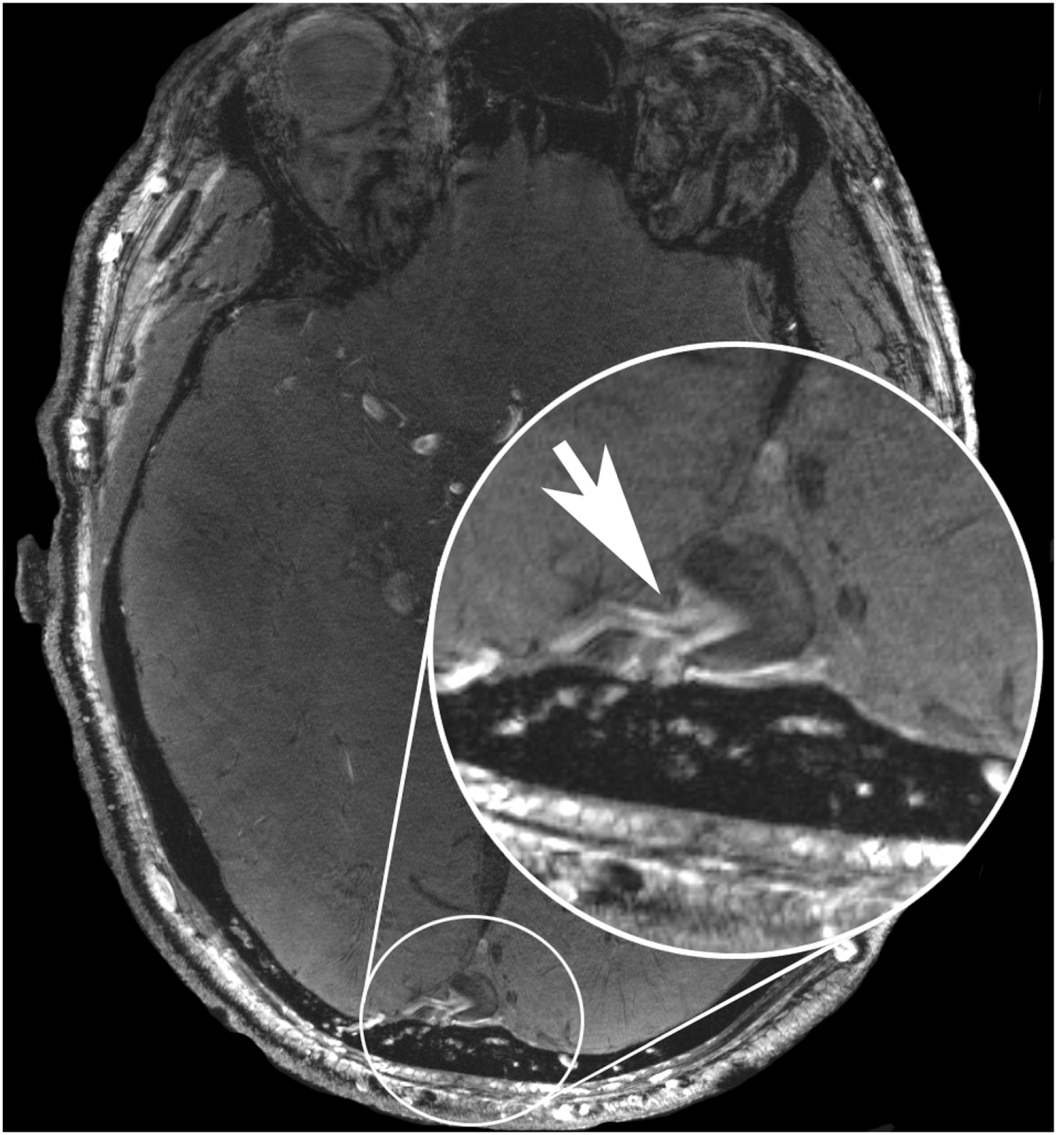
This figure shows the scan of a 58-year-old male patient (subject 11) with a complex occipital dural AVF (Cognard type IIb) fed by branches of both external carotid and vertebral arteries. It highlights the advantages of venous saturation pulses in 7 Tesla TOF MRA. The exact location of the fistulous point (arrow in magnified image section) is depicted by mixing hyperintense unsaturated arterial blood with hypointense saturated venous blood in the superior sagittal sinus.

Two patients with arteriovenous fistulas were enrolled in the study (male n = 2, female n = 0; average age 59.5 years; range 58–61 years). Consensus ratings were excellent for delineation of Circle of Willis (mean 5.0, range 5–5, SE 0.0), moderate and good for vascular pathology delineation (mean 3.5, range 3–4, SE 0.5), moderate for vessel-tissue contrast (mean 3.0, range 3–3, SE 0.0), good for presence of artifacts (mean 4.0, range 4–4, SE 0.0) and good for overall image quality (mean 4.0, range 4–4, SE 0.0). The applied venous saturation pulses resulted in vessel-tissue contrast ratios as follows: mean VTCR_CoS = 0.131 (range 0.121–0.141, SE 0.01); VTCR_MCA = 0.678 (range 0.628–0.728, SE 0.05). As in AVM patients venous saturation was lower compared to other pathologies due to inherent features of dural AVFs, in particular direct shunting of arterialized blood into the venous sinuses. High signal intensity of arterialized blood is similar to signal intensity of the left middle cerebral artery M1 segment. Signal intensity of venous sinuses decays the further away from the fistula point it is measured. The special features of dural AVF in 7 Tesla TOF MRA scans are exemplarily illustrated (Figure 5) in a 58-year-old male patient (subject 11) with a complex occipital dural AVF (Cognard [32] type IIb) fed by branches of both external carotid and vertebral arteries.

## Discussion

Time-of-flight MRA is a non-invasive imaging technique using FLASH gradient-echo magnetic resonance sequences to visualize the contrast between the high signal intensity of inflowing blood and saturated stationary tissue. TOF MRA utilizing 1.5 Tesla has been established as an excellent clinical diagnostic method for pre-operative and pre-interventional planning as well as for follow-up controls in cerebro-vascular pathologies, and has gained ground on the accepted gold standard, invasive digital subtraction angiography.

The increase of the magnetic field strength from 1.5 Tesla to 3 Tesla has been proven beneficial for clinical neuroimaging and feasible for ultra-high-field 7 Tesla MRI, facilitating a significant increase in spatial resolution [4], [8], [9], [15]–[17], [33], [34]. Moenninghoff et al. presented a high-resolution 7 Tesla TOF MRA protocol for clinical application including venous saturation pulses. To maintain SAR limitations, time-of-repetition (TR) had to be increased up to 57 ms, resulting in an increase in acquisition time to almost 15 minutes for comparable spatial coverage as the presented protocol. The previously suggested solution [27] to overcome SAR limitations within a patient tolerable scanning time has not yet been applied in a clinical setting for various intracerebral vascular pathologies.

The impairment of venous saturation at ultra-high field strength is mainly due to SAR limitations that prohibit the usage of venous saturation pulses within reasonable acquisition times.

One proposed solution to overcome SAR limitations is to play out a regular 90° saturation pulse only every n-th readout of the k-space [8]. The drawback of this approach would be that either measurement time is not used efficiently or excitation RF pulses experience different effective TRs which could lead to an artifact appearing similar to aliasing [35] caused by periodic signal variation in k-space lines. The exact effect of different effective TRs would have to be assessed prior to routine clinical application. It is also difficult to achieve a good balance between venous saturation and optimal SAR utilization, as this method is more rigid than mere flip angle variations. Our approach to overcome SAR limitations uses customized low flip angle VERSE algorithm [28], [29] saturation pulses to significantly reduce the SAR of each individual saturation pulse rather than reducing the number of saturation pulses. The VERSE algorithm was used to decrease SAR contribution of excitation and saturation RF pulses. Based on this reduction, flip angles of saturation pulses were optimized for adequate venous saturation while remaining within SAR restrictions. Without optimization of flip angles and VERSE algorithm acquisition time was almost 3 times longer (6∶22 min versus 17∶48 min) in a 29-year-old healthy volunteer and TE had to be increased from 4.34 ms to 6.62 ms. Also the TR had to be increased from 20 ms to 56 ms to stay within SAR limits. Especially the prolonged TR leads to lower image contrast. A detailed description of the applied pulse sequence and considerations on ideal flip angles for saturation pulses have previously been published [27]. Differences in image contrast between non-optimized and optimized TOF MRA sequences are illustrated in Figure 1.

Only in patients with AVMs and AVF, venous saturation was reduced compared to other pathologies. This was presumably due to the inherent features of both pathologies, specifically the fast blood flow of venous drainage. In addition, only part of the AVMs were within the field of saturation pulses and consequently unsaturated blood was drained over the veins. Even though the venous saturation was reduced in these patients, the application of saturation pulses could still partially suppress the signal of draining veins with an overall good image quality.

The high diagnostic relevance of saturation pulses in combination with ultra-high-resolution TOF MRA is exemplarily shown in the case of Subject 12 (Figure 5). The complex dural AVF (Cognard [32] type IIb) could be clearly visualized and the fistulous point with the hyperintense artery entering the hypointense venous sinus was clearly depicted by mixed unsaturated arterial and saturated venous blood. This effect can presumably only be visualized with effective venous saturation, and might not be clearly visible in TOF MRA imaging without venous saturation pulses or in contrast-enhanced T_1_-weighted images. Future patients suffering of AVF will be examined with and without venous saturation pulses to further investigate the diagnostic potential of this effect.

Consensus ratings of two experienced readers ranged between good and excellent for the majority of patients and most of the evaluated features. In combination with short acquisition time and possibility to visualize the complete intracranial arterial vasculature with ultra-high resolution the presented TOF MRA protocol is suitable for clinical application.

Clearly, this study is not free of limitations. The study group comprised of patients suffering from various intracranial vascular pathologies and therefore patient numbers for individual pathologies are small. Nevertheless, to the authors knowledge this is one of the largest neurosurgical patients cohorts scanned with TOF MRA at 7 Tesla, published in literature. Further investigations with larger patient cohorts of single pathologies should be the focus of future studies. Ultra-high-field imaging in patients who underwent treatment has been restricted so far, as Guglielmi detachable coils, aneurysm clips, most cranial fixation plates and other metallic implants are not certified for 7 Tesla MR imaging. Although preliminary results on implant safety in cerebral 7 Tesla MRI have been recently demonstrated [36]–[38], up to now avoiding metallic implants during surgery is the only feasible way for postoperative scans. The excellent image quality is exemplarily shown in a 42-year-old female patient (subject 25) after bilateral superficial temporal artery to middle cerebral artery bypass surgery (Figure 4). Future studies on the diagnostic potential of 7 Tesla MRI for follow-up of treated aneurysms, AVM, AVF and patients with Moyamoya angiopathy would be of high scientific and clinical interest. Another limitation is posed by the lack of a comparison to digital subtraction angiography, the diagnostic gold standard. However, with MRA offering equivalent non-invasive vessel diagnostics to DSA, the main focus of this trial was set on testing the feasibility and diagnostic potential of high-resolution TOF MRA at 7 Tesla utilizing novel low flip angle venous saturation pulses in a variety of intracranial vascular pathologies.

In summary, this study presents the clinical application of the previously reported practical solution for saturation pulses in 7 Tesla ultra-high-resolution TOF MRA that maintains SAR levels within the accepted safety standards combined with short acquisition times [27]. Excellent imaging quality was shown for the majority of patients in a larger cohort suffering from diverse cerebro-vascular pathologies.
